# Internet Gaming Addiction in Male Adolescents: Mitochondrial DNA Variations and Leukocyte Telomere Length

**DOI:** 10.3390/genes17080859

**Published:** 2026-07-24

**Authors:** Nahyun Kim, Jooyeon Park, In Deok Kong, Tonda L. Hughes, Dae-Kwang Kim

**Affiliations:** 1 College of Nursing, Keimyung University, Daegu 42601, Republic of Korea; drkim@kmu.ac.kr; 2 College of Nursing, Daegu University, Daegu 42400, Republic of Korea; jypark1185@daegu.ac.kr; 3 Wonju College of Medicine, Yonsei University, Wonju 26426, Republic of Korea; kong@yonsei.ac.kr; 4 School of Nursing, Columbia University, New York, NY 10032, USA; th2696@cumc.columbia.edu; 5 School of Medicine, Keimyung University, Daegu 42601, Republic of Korea

**Keywords:** internet gaming addiction, mitochondrial DNA, D-loop, polymorphism, copy number, telomere length

## Abstract

Background/Objectives: Mitochondrial DNA (mtDNA) variations are linked to psychiatric disorders, but their association with internet gaming addiction (IGA) remains unexplored. We investigated mitochondrial D-loop D310 and D514 regions and mtDNA copy number (mtCN) in male adolescents with and without IGA, exploring their associations with leukocyte telomere length (LTL). Methods: Questionnaires assessed IGA in 206 male adolescents. Blood samples were analyzed for (C)_n_ repeats at D310 and (CA)_n_ repeats at D514. Relative mtCN and LTL were measured using quantitative PCR. Results: D310 polymorphism distribution differed significantly between the IGA and non-IGA groups (*p* = 0.040). Among the (C)_n_ repeats, (C)_8_ frequency at D310 was significantly lower in the IGA group than in the non-IGA group (*p* = 0.017). Multivariable logistic regression initially identified the (C)_8_ polymorphism as an independent predictor reducing IGA likelihood (*OR* = 0.474, *p* = 0.014), although this nominal association did not remain statistically significant after formal Bonferroni correction. mtCN differences were not significant (*p* = 0.247). Notably, LTL was significantly shorter in the IGA group among carriers of (C)_8_ and (CA)_5_ polymorphisms (both *p* = 0.001). Multivariable linear regression confirmed that IGA remained robustly associated with shorter LTL (*B* = −40.180, *p* < 0.001), while (C)_8_ and (CA)_5_ repeats were not independently associated with LTL shortening. Conclusions: While the (C)_8_ polymorphism’s direct genetic contribution to IGA susceptibility remains preliminary and hypothesis-generating due to multiple testing attenuation, IGA exhibits a robust, independent association with LTL shortening. Mitochondrial genomic backgrounds may play a subtle, modulatory role in behavioral addiction pathways, warranting further longitudinal validation.

## 1. Introduction

Internet gaming disorder (IGD) was introduced as a formal diagnosis by the World Health Organization (WHO) in the 11th revision of the International Classification of Diseases (ICD-11), under disorders due to substance use or addictive behaviors [1]. IGD was also identified in the Diagnostic and Statistical Manual of Mental Disorders, 5th Edition (DSM-5), as a condition warranting further research [2]. Consequently, IGD, also referred to as internet gaming addiction (IGA), has been widely investigated with respect to associated personality traits, psychosocial factors, and neurobiological mechanisms, and is increasingly recognized as a mental disorder. Nevertheless, its inclusion in ICD-11 remains controversial because of insufficient empirical evidence, highlighting the need for further research.

A recent national survey reported that 99.1% of Korean adolescents access the internet daily, 58.8% play personal computer games, and 82.4% engage in mobile gaming [3]. Given that health-related behaviors established during adolescence can have profound public health implications [4], excessive internet gaming in this population has become a major societal concern.

Genetic variations have been implicated in addictive disorders, including IGA, with evidence suggesting that persistent addictive behaviors may induce alterations in gene expression that contribute to the development of addictive phenotypes [5,6,7]. In addition, leukocyte telomere length (LTL) has been identified as an independent biological marker in several psychiatric disorders [8,9]. Despite these findings, research focusing on the biological and genetic underpinnings of IGA remains limited [10]. In recent years, mitochondrial DNA (mtDNA) variations have gained attention as potential biomarkers in the pathophysiology of numerous diseases [11,12]. Compared with nuclear DNA (nDNA), mtDNA is particularly susceptible to oxidative damage due to limited DNA repair capacity and the absence of protective histones [13].

The mitochondrial displacement loop (D-loop) is a noncoding region that regulates mtDNA replication and transcription [14] and exhibits a higher mutation rate than other mtDNA regions [13,14,15]. Within the D-loop, the D310 and D514 regions are well-established hotspots for genetic variation [16,17]. In normal human tissues, D310 contains cytosine repeat polymorphisms ranging from (C)_6_ to (C)_9_, whereas D514 comprises cytosine–adenosine repeat polymorphisms ranging from (CA)_3_ to (CA)_7_ [18]. Variations in the D-loop can influence mtDNA transcription and impair respiratory chain function, thereby disrupting mitochondria-mediated apoptosis and oxidative metabolism [19,20]. Accordingly, D-loop polymorphisms have been associated with the onset and progression of various diseases [15,17,21]. Moreover, mtDNA copy number (mtCN), an index of mitochondrial biogenesis and mtDNA content [22], is considered a reliable biomarker of mitochondrial dysfunction, as alterations in mtCN are closely linked to changes in mitochondrial function [23,24,25,26].

Interactions between mitochondria and telomeres play a critical role in cellular senescence, age-related disease development [24], carcinogenesis [7,23], and psychiatric disorder onset [25,26]. Telomere dysfunction can impair mitochondrial biogenesis and energy production [24,25,27], leading to increased reactive oxygen species (ROS) generation, reduced ATP levels, and subsequent DNA damage. Furthermore, mitochondrial dysfunction may induce telomere attrition [27]. Stress-related allostatic processes have been shown to affect both telomeres and mitochondria through shared mechanisms, further supporting a functional link between these systems [28].

To date, the association between IGA and mtDNA variations has not been investigated. In a previous study, LTL was significantly shorter in adolescents with IGA than in those without IGA [8]. Given that LTL has been reported to show both positive and negative associations with mtDNA parameters [9,23,24,25,26], IGA may be associated with accelerated alterations in mitochondrial biogenesis. Therefore, in the present study, we aimed to examine differences in mtDNA D-loop polymorphisms and mtCN in circulating leukocytes between Korean male adolescents with and without IGA. In addition, we investigated the associations between mtDNA variations and LTL across both study groups. 

## 2. Materials and Methods

### 2.1. Study Design

This study was a part of a large cross-sectional investigation examining the roles of autonomic function and genetic factors in the development of IGA among male adolescents.

### 2.2. Participants and Sample Procedures

**Figure 1:**
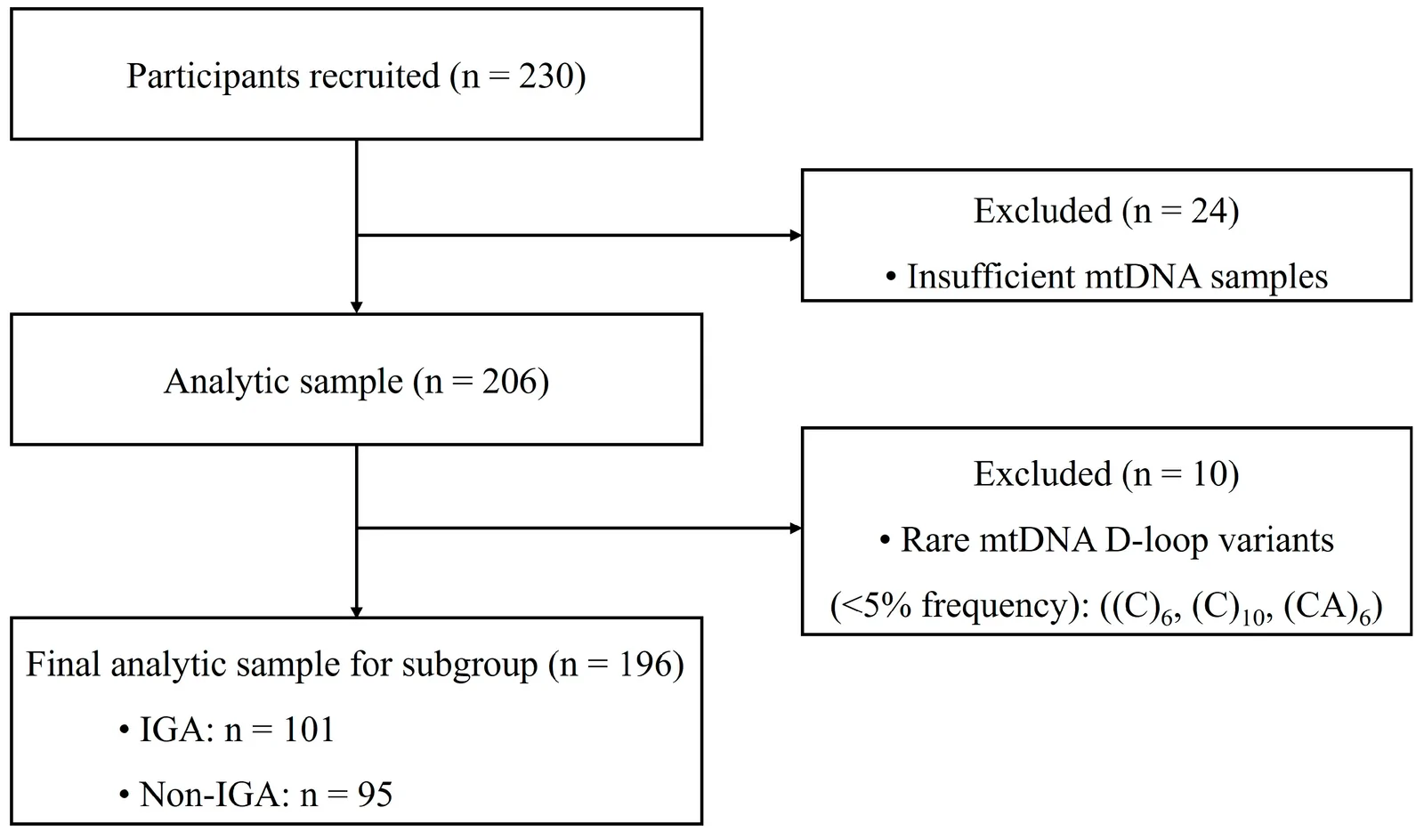
Flow diagram of the participant selection process.

A total of 230 male high school students were recruited from a community center in South Korea using convenience and snowball sampling methods, as previously described [29]. All participants completed self-administered questionnaires in a private setting and provided blood samples. Data from 24 participants were excluded because of insufficient mtDNA samples, resulting in an initial sample of 206 individuals for demographic characterization. For the subsequent genetic association analyses of mtDNA D-loop polymorphisms (D310 and D514), 10 additional participants were excluded due to the presence of rare variants with a frequency of less than 5% (including one case of overlap). This exclusion was necessary to ensure the statistical robustness and reliability of the categorical comparisons, leaving a final analytic sample of 196 individuals for the polymorphism analyses. The detailed recruitment and exclusion process is presented in the participant flow diagram (Figure 1). The mean age of the participants was 16.66 ± 1.02 years. Approximately 35% of participants in both the IGA and non-IGA groups reported smoking, alcohol consumption, or both. Daily sleep duration differed significantly between the groups (*p* = 0.033). The mean duration of internet gaming experience was significantly longer in the IGA group than in the non-IGA group (7.76 ± 2.35 vs. 6.78 ± 2.38 years, respectively; *p* = 0.003). Additional demographic characteristics and internet gaming-related variables have been previously reported [8]. The study protocol was approved by the Institutional Review Board of Y University (Ref: YWMR-12-0-015). Written informed consent was obtained from all participants and their legal guardians prior to data collection.

### 2.3. Measurements

#### 2.3.1. IGA

IGA was evaluated using the Internet Gaming Addiction (IGA) Scale for Adolescents, developed by the Korean Agency for Digital Opportunity and Promotion [30]. The original 20-item self-report questionnaire demonstrated high internal consistency with a Cronbach’s alpha = 0.93 and was validated by a panel of 20 experts to ensure content validity. It has served as a national standard for screening at-risk youth in South Korea. The scale employs a 4-point Likert scale, with higher scores indicating greater severity of IGA, as previously described [8,29]. Although the scale was developed prior to the formalization of the current diagnostic framework, its items are structurally aligned with the core criteria for IGD as defined in the DSM-5 and ICD-11. Specifically, the instrument comprehensively assesses core symptoms, including preoccupation, withdrawal, tolerance, and functional impairment in daily life. Given its high linguistic and cultural validity for the Korean adolescent population, this instrument effectively captures the behavioral and clinical characteristics of IGA. For analysis, participants were classified into either the IGA group (total scores ≥ 38) or the non-IGA group (total scores < 38) based on the standardized cut-off points.

#### 2.3.2. DNA Extraction

Genomic DNA was extracted from the blood samples of all 230 male participants. Whole blood was collected in EDTA-containing tubes and stored at −20 °C until processing. The samples were thawed prior to extraction, and DNA was isolated using the QIAamp Blood Kit (Qiagen, Hilden, Germany) following the manufacturer’s instructions. The extracted DNA was eluted in 50 μL of Tris-EDTA buffer (10 mM Tris-HCl, 1 mM EDTA, pH 8.0).

#### 2.3.3. Direct DNA Sequencing of the Polymerase Chain Reaction Products

Mitochondrial D-loop polymorphisms were analyzed by amplifying two target regions: the (C)_n_ repeat at position 310 and the (CA)_n_ repeat at position 514. The primers used were as follows: D310: forward 1F, 5′-TCCACACAGACATCATAACA-3′; reverse 1R, 5′-AAAGTGCATACCGCCAAAAG-3′; D514: forward 2F, 5′-CCCATATCTAATCTCAA-3′; reverse 2R, 5′-TTTGGTTGGTTCGGGGTATG-3′. Polymerase chain reaction (PCR) was performed using a thermal cycler (Model 2400, Applied Biosystems, Waltham, MA, USA) under the following conditions: 35 cycles of denaturation at 94 °C for 40 s, annealing at 56 °C for 40 s, and extension at 72 °C for 40 s, followed by a final extension at 72 °C for 10 min. The PCR products were resolved by agarose gel electrophoresis and visualized with ethidium bromide to confirm fragment sizes. Direct DNA sequencing of the PCR products was subsequently performed to identify the (C)_n_ and (CA)_n_ polymorphisms within the mitochondrial D-loop.

#### 2.3.4. Quantitative PCR

Quantitative PCR (qPCR) using SYBR Green dye was performed to measure mtCN and LTL via an established and validated qPCR-based technique [8]. For the results presented herein, qPCR amplification was performed as described by Cawthon [31] and Imam and colleagues [32] using a Thermal Cycler Dice Real Time System II (TP850; Takara Bio Inc., San Jose, CA, USA) operated with Thermal Cycler Dice Real Time System Single Software (Version 5.11B) with the following modifications. To quantify mtDNA content relative to nDNA, primers were designed to specifically amplify the mitochondrially encoded *COX1* gene and the single-copy nDNA-encoded human *β*-globin (*HGB3* reference gene). The *COX1* primers were 5′-CCCCACATTAGGCTTAAAACCAGAT-3′ (forward) and 5′-TATACCCCCGGTCGTGTAGC-3′ (reverse), while the *HGB3* primers were 5′-GCTTCTGACACAACTGTGTTCACTAGC-3′ (*HGB3 F*) and 5′-CACCAACTTCATCCACGTTCACC-3′ (*HGB3 R*). To measure LTL, the telomere-specific primers were 5′-CGGTTTGTTTGGGTTTGGGTTTGGGTTTGGGTTTGGGTT-3′ (*Tel F*) and 5′-GGCTTGCCTTACCCTTACCCTTACCCTTACCCTTACCCT-3′ (*Tel R*), as previously described [8]. The SYBR Green qPCR was divided into separate reactions: target region PCRs (*COX1* and telomere) and single-copy gene PCRs (*HGB3*). Each 20-μL reaction mixture consisted of 1× SYBR Green I Master Mix (Roche, Basel, Switzerland), 10 μM (final concentration: 0.4 μM) of each primer, and 10 ng of genomic DNA. The same thermal cycling conditions were applied to all targets (*COX1*, *HGB3*, and telomeres) as follows: initial denaturation at 95 °C for 10 min, followed by 40 cycles of denaturation at 95 °C for 10 s, annealing at 59 °C for 10 s, and extension at 72 °C for 10 s. Standard and melting curves were generated for *COX1*, *HGB3*, and telomere primers to check the amplification efficiency and specificity of the qPCR assays, respectively. All measurements were strictly performed in triplicate.

Relative LTL and mtCN were determined using the 2^−∆∆*C*^*^t^* method. For individual samples, ∆*Ct* for LTL was calculated as (*Ct*,*_Tel_* − *Ct*,*_HGB3_*), and ∆*Ct* for mtCN was calculated as (*Ct*,*_COX1_* − *Ct*,*_HGB3_*). The relative LTL was calculated as the T/S ratio, which is the ratio between telomere repeat length and the copy number of the single-copy gene (*HGB3*), determined using the formula [2*^Ct^*^,*Tel*^/2*^Ct^*^,*HGB3*^]^−1^ = 2^−Δ*Ct*^. Subsequently, the relative fold-change was derived by calculating ∆∆*Ct*, which was determined by subtracting the average ∆*Ct* of the non-IGA reference group from the ∆*Ct* of each individual sample (∆∆*Ct* = ∆*Ct*,*_sample_* − ∆*Ct*,*_non-IGA_mean_*), in accordance with the established real-time PCR quantification framework [31]. The intra-assay coefficient of variation (CV) was <2% for all qPCR measurements, including *COX1* (0.59%), *HGB3* (0.65%), and telomeres (1.66%), indicating high reproducibility of the assays.

### 2.4. Data Analysis

All statistical analyses were conducted using SPSS version 22.0 (IBM, Armonk, NY, USA). Baseline characteristics of participants are presented as frequency, percentage, mean, and standard deviation. Differences in mtDNA variations according to baseline characteristics were assessed using the χ^2^ test, independent two-sample *t*-test, or one-way analysis of variance, as appropriate. Comparisons of D-loop polymorphisms and mtCN between the IGA and non-IGA groups were conducted using the χ^2^ test and independent *t*-test. Associations between mtCN and LTL were examined using Pearson’s correlation coefficient. Relative LTL was calculated as the T/S ratio, as previously described [8]. To examine factors associated with IGA, multivariable logistic regression analysis was performed, adjusting for potential confounders including smoking, alcohol consumption, and sleep duration. Odds ratios (ORs) and 95% confidence intervals (CIs) were calculated. In addition, to further evaluate the association between mtDNA polymorphisms and LTL, multivariable linear regression analysis was conducted, adjusting for the same covariates. Interaction terms between IGA and mtDNA polymorphisms were included to explore potential effect modification. Statistical significance was initially set at *p* < 0.05. Because multiple comparisons were performed across various genetic polymorphisms and biomarkers, formal Bonferroni corrections were additionally applied to mitigate the risk of Type I error. For the multivariable logistic regression analysis, the significance threshold was adjusted to *p* < 0.01 (0.05 divided by 5 polymorphism comparisons). For the multivariable linear regression analysis, the significance threshold was adjusted to *p* < 0.008 (0.05 divided by 6 independent comparisons). Prior to model interpretation, the mathematical assumptions underlying the parametric analyses, including the normality and homoscedasticity of residuals, were formally assessed and satisfied.

## 3. Results

Table 1 presents the genotype distributions of mtDNA D-loop polymorphisms and mtCN in the study groups. We identified five (C)_n_ repeat alleles at the D310 site, ranging from (C)_6_ to (C)_10_, and three (CA)_n_ repeat alleles at D514, ranging from (CA)_4_ to (CA)_6_. The most prevalent sequences were (C)_7_ at D310 and (CA)_5_ at D514. Notably, the distribution of D310 polymorphisms showed a significant difference between the IGA and non-IGA groups (*p* = 0.040). In the non-IGA group, the (C)_8_ variant was the most frequent (45.5%), whereas the (C)_7_ variant predominated in the IGA group (41.9%). In contrast, for D514 polymorphisms, the (CA)_5_ variant was the most common in both the non-IGA and IGA groups (61.4% and 57.1%, respectively), and no significant difference in the distribution of (CA)_n_ repeat polymorphisms was observed (*p* = 0.525). Regarding mtCN, although the IGA group exhibited an approximately 10% lower mtCN than the non-IGA group, this difference did not reach statistical significance (*p* = 0.247), indicating a lack of association between mtCN and IGA in this study population.

Table 2 summarizes the differences in mtDNA D-loop polymorphisms and mtCN according to the baseline characteristics of the participants. The distribution of (C)_n_ repeat polymorphisms did not differ significantly by age, smoking, alcohol consumption, or sleep duration. In contrast, the distribution of (CA)_n_ repeat polymorphisms differed significantly according to smoking status (*p* = 0.010). Furthermore, mtCN was significantly lower in participants who smoked (*p* = 0.020) and those who consumed alcohol (*p* < 0.001) than in non-smokers and non-drinkers. In the total study population, we observed no significant associations between mtDNA D-loop polymorphisms and mtCN or LTL. Specifically, neither (C)_n_ nor (CA)_n_ repeat polymorphisms significantly correlated with mtCN (*p* = 0.289 and *p* = 0.053, respectively) or LTL (*p* = 0.487 and *p* = 0.289, respectively). In addition, mtCN did not show a significant correlation with LTL (*p* = 0.318).

Among the (C)_n_ repeat polymorphisms, the frequency of the (C)_8_ variant was significantly lower in the IGA group than in the non-IGA group (*p* = 0.017). However, this nominal association did not remain statistically significant after the formal Bonferroni correction for multiplicity across the five polymorphism comparisons, which utilized an adjusted significance threshold of *p* < 0.01 (0.05/5). In addition, there was no significant difference between the groups in terms of the distribution of (CA)_n_ repeat polymorphisms (all *p* > 0.05; Table 3).

To account for potential confounding variables, a multivariable logistic regression analysis was performed. Variables initially included in the model were smoking, alcohol consumption, sleep duration, and mtDNA D-loop polymorphisms ((C)_8_ and (CA)_5_). In the final model, the (C)_8_ polymorphism and sleep duration remained a significant predictor of IGA. Crucially, adolescents carrying the (C)_8_ polymorphism were significantly less likely to belong to the IGA group than those without the polymorphism (*OR* = 0.474; 95% *CI*: 0.262 to 0.858; *p* = 0.014). In addition, longer sleep duration was associated with a lower likelihood of IGA (*OR* = 0.519; 95% *CI*: 0.280 to 0.965; *p* = 0.038). However, this nominal association did not remain statistically significant after the formal Bonferroni correction for the five polymorphism comparisons, which utilized an adjusted significance threshold of *p* < 0.01 (0.05/5). Smoking, alcohol consumption, and the (CA)_5_ polymorphism was not significantly associated with IGA in the final adjusted analysis. The overall model was statistically significant (*χ*^2^ = 10.133, *p* = 0.006), with a Nagelkerke *R*^2^ of 0.067 and an overall classification accuracy of 58.7% (Table 4).

We further compared mtCN and LTL between the IGA and non-IGA groups according to mtDNA D-loop polymorphisms. Across all polymorphisms, except for (C)_9_, mtCN was lower in the IGA group than in the non-IGA group. However, none of these differences reached statistical significance. With respect to LTL, it was significantly shorter in the IGA group than in the non-IGA group only among adolescents carrying the (C)_8_ and (CA)_5_ polymorphisms (both *p* = 0.001). In contrast, no significant differences in LTL were observed between the groups among adolescents with other D-loop polymorphisms (Table 5).

To further examine the independent association between mtDNA D-loop polymorphisms and LTL, a multivariable linear regression analysis was conducted adjusting for smoking, alcohol consumption, and sleep duration (Table 6). The overall model was statistically significant (*F* = 3.717, *p* = 0.002), explaining 10.6% of the variance in LTL (*R*^2^ = 0.106). IGA was strongly and significantly associated with LTL after adjustment, with adolescents in the IGA group showing shorter LTL compared with the non-IGA group (*B* = −40.180, *p* < 0.001). This association remained statistically significant after the formal Bonferroni correction for the six independent comparisons, which utilized an adjusted significance threshold of *p* < 0.008 (0.05/6). In contrast, mtDNA D-loop polymorphisms, including (C)_8_ and (CA)_5_, were not significantly associated with LTL (*p* = 0.639 and *p* = 0.222, respectively). Smoking (*p* = 0.121) and alcohol consumption (*p* = 0.096) showed trends toward associations with LTL but did not reach statistical significance. Sleep duration was also not significantly associated with LTL.

## 4. Discussion

In this study, we examined whether IGA is associated with mitochondrial variations, as reflected by mtDNA D-loop polymorphisms and mtCN. We also evaluated the relationship between mitochondrial variations and LTL in adolescents with and those without IGA.

The observed range of cytosine repeat polymorphisms at the D310 region of the mtDNA D-loop was (C)_6_ to (C)_10_, whereas the cytosine–adenosine repeat polymorphisms at the D514 region ranged from (CA)_4_ to (CA)_6_. Most of these polymorphisms were within the previously reported normal ranges [16,17,18], suggesting that the majority of mtDNA variations identified in this study are unlikely to directly impair mitochondrial function [15]. Notably, 1.5% of D310 variants consisted of the relatively rare (C)_10_ repeat, which may reflect ethnic differences in D-loop polymorphism distributions [15,18].

Regarding the relationship between mtDNA D-loop polymorphisms and IGA, we observed a significant difference in the distribution of D310 polymorphisms between the IGA and non-IGA groups. The D310 region is a known hotspot for pathogenic mutations, and variation in the number of cytosine repeats is considered a marker of mtDNA instability [17]. Among the (C)n repeats at D310, the frequency of the (C)_8_ polymorphism was significantly lower in the IGA group than in the non-IGA group. In contrast, no significant group differences were observed in the distribution of D514 polymorphisms. Although most D-loop polymorphisms are unlikely to cause overt mitochondrial dysfunction [15], they have been reported to serve as important susceptibility biomarkers for a range of diseases [15,33,34]. Collectively, our results indicate that mtDNA D-loop polymorphisms may be associated with IGA, with the (C)_8_ repeat at D310 emerging as a notable variant within this context. 

In the adjusted multivariable logistic regression model, the (C)_8_ repeat was initially identified as a significant independent predictor of IGA likelihood, although this nominal association did not remain statistically significant after formal Bonferroni correction. Nevertheless, this preliminary trend suggests that mitochondrial genomic backgrounds may still warrant cautious exploration as potential indirect modulators in larger, independent replication cohorts. 

In our previous study, LTL was significantly shorter in male adolescents with IGA than in those without IGA [8]. In the present study, univariate analysis similarly demonstrated that LTL was notably shorter in adolescents with IGA who carried the (C)_8_ and (CA)_5_ repeat polymorphisms. To further validate these associations and extend our previous findings by controlling for confounding factors, we conducted a multivariable linear regression analysis adjusting for potential confounders such as smoking, alcohol consumption, and sleep duration. The results revealed that IGA remained a strong and significant independent factor associated with LTL shortening (*B* = −40.180, *p* < 0.001), confirming a highly robust and independent relationship. However, when concurrently analyzed within the regression model, both (C)_8_ and (CA)_5_ repeat polymorphisms were not significantly associated with LTL shortening. These collective findings require a nuanced adjustment of our interpretation. Rather than exerting a direct causal effect on LTL, these specific D-loop polymorphisms might serve as a subclinical mitochondrial genomic background or a placeholder for general genetic vulnerability. When an individual carrying this particular genomic background is chronically exposed to the severe psychosocial stress and sedentary lifestyle associated with addictive gaming behaviors, it may synergistically facilitate early biological alterations and genomic instability, ultimately accelerating cellular aging in this population. 

Taken together, these observations indirectly support the hypothesis that mtDNA D-loop polymorphisms may be linked to the baseline biological characteristics of individuals vulnerable to IGA, albeit strictly at a speculative level. While we caution against overinterpreting these unmeasured mechanistic pathways in the context of IGA, we hypothesize that genetic variability within the mitochondrial D-loop region could potentially influence respiratory chain function, which may lead to increased ROS production and subsequent damage to the nuclear genome [21,35]. Although the present study did not directly measure oxidative stress markers or mitochondrial respiratory activity, this preliminary hypothesis is conceptually supported by previous reports suggesting that D-loop variations can disrupt transcription factor binding and induce potential electron leakage from the respiratory chain [36]. Nevertheless, we explicitly present these potential pathways as preliminary hypotheses for future functional validation rather than confirmed mechanistic links in IGA. 

In addition, we observed that mtCN was significantly lower in adolescents who smoked or consumed alcohol, consistent with the findings of previous studies [12,37]. In our population, however, mtCN did not significantly differ between the IGA and non-IGA groups, indicating a lack of association between mtCN and IGA. Moreover, mtCN was not significantly associated with LTL in our study population. These findings contrast with reports of altered mtCN in certain psychiatric disorders, such as anxiety and depression [25,26], but are consistent with reports of no association between mtCN and psychiatric conditions [9]. The absence of a significant difference in mtCN suggests that mitochondrial genomic abundance may not be a primary feature of IGA pathology, at least within the age range of our participants. The reports regarding mtCN alterations in psychiatric and medical disorders remain inconsistent, particularly with respect to causal relationships. Therefore, further studies with larger cohorts and longitudinal designs are warranted to delineate these associations.

Overall, our findings suggest that associations among IGA, mitochondrial D-loop polymorphisms, and telomere shortening may be linked to excessive internet gaming behavior at a strictly hypothetical level. However, IGA does not appear to be associated with overt mitochondrial dysfunction, as evidenced by the lack of significant differences in mtCN. This observation is consistent with the findings of a 10-year longitudinal study, which reported telomere shortening in individuals with depressive symptoms in the absence of concomitant reductions in mtCN [9]. One possible explanation for the lack of a significant association between IGA, mtCN, and LTL may relate to both gaming duration and the relatively young age of the participants. Although adolescents in the IGA group reported longer gaming durations than those in the non-IGA group (7.76 vs. 6.78 years, respectively), the non-IGA group also engaged in internet gaming for a substantial period. Therefore, while mtCN in the IGA group was lower than that in the non-IGA group, the difference may not have reached statistical significance in this specific cohort. While such behavioral exposure may contribute to telomere shortening—as robustly reinforced by our multivariable regression results—it may be insufficient to induce measurable mitochondrial dysfunction at this stage [7,24,25,26]. The aforementioned longitudinal study demonstrated that participants with depressive symptoms exhibited relatively stable LTL during the first 5 years of follow-up, followed by a marked decrease in LTL during the subsequent 5 years [9]. By analogy, mitochondrial dysfunction may emerge later in the course of the disease rather than at the initial onset of a pathophysiological condition. Age may also be a contributing factor, as mitochondrial damage is less likely to manifest in younger individuals [9,25,38,39]. 

Although our results do not provide direct evidence for a telomere–mitochondrial axis in IGA, they suggest that the underlying mechanisms linking telomeres and mitochondria may be complex and age dependent. In healthy young individuals, mitochondrial resilience may attenuate detectable mitochondrial damage despite the presence of telomere shortening [25,39]. In summary, while the (C)_8_ polymorphism was linked to LTL shortening strictly within the univariate stratification of the IGA group, this genetic variation did not lead to overt mitochondrial dysfunction in this adolescent population—a finding that should be interpreted in light of several study limitations. 

This study had several limitations. First, the cross-sectional design precludes causal inferences regarding the relationships between IGA, D-loop polymorphisms, and LTL shortening. Therefore, longitudinal studies are needed to confirm and extend these preliminary findings. Second, although we adjusted for core lifestyle factors (smoking, alcohol and sleep), other critical confounding variables—such as body mass index, objective physical activity intensity, psychiatric comorbidities, socioeconomic status, and total daily screen time—were not directly evaluated and remain potential sources of residual confounding. Third, mtDNA measurements were obtained from mixed leukocyte populations, and variation in cell-type composition may have influenced the results. Nevertheless, previous research has shown that mtDNA profiles derived from whole blood are comparable to those obtained from isolated lymphocytes [40]. Fourth, IGA was assessed using a self-report questionnaire, which captures behavioral addiction severity but does not replace a structured clinical diagnostic assessment for gaming disorder. Therefore, caution is warranted when extrapolating these biological correlates to clinically diagnosed patient populations. Additionally, although the study population was ethnically homogeneous, mitochondrial haplogroup structure was not considered, which may have influenced the observed associations. Fifth, the multivariable logistic regression model exhibited low explanatory power (Nagelkerke *R*^2^ = 0.067), indicating that the majority of the variability in IGA status remains unexplained by the included predictors. Sixth, while the sample size was sufficient for our primary LTL regression model, statistical power may have been insufficient for capturing subtle genetic association effects after stringent multi-testing penalties. Finally, this study involved multiple comparisons across various genetic polymorphisms and biomarkers, potentially increasing the risk of Type I error. To mitigate this concern and rigorously evaluate the robustness of our data, we formally implemented Bonferroni adjustments across our multivariable models, which revealed that the initial genetic association regarding the (C)_8_ variant did not survive this stringent threshold. Consequently, as demonstrated by these adjusted analyses, our findings—especially those involving the (C)_8_ polymorphism—must be interpreted with extreme caution. Given the exploratory and strictly hypothesis-generating nature of these genetic associations, further replication in independent, larger cohorts is still required to definitively validate our preliminary observations and fully elucidate the underlying pathways.

## 5. Conclusions 

In summary, the initial analysis demonstrated that the mtDNA D-loop (C)_8_ repeat polymorphism shows only a nominal association with a reduced likelihood of IGA, rather than serving as a definitive independent predictor, as the association did not survive strict Bonferroni correction. Given that this preliminary, hypothesis-generating signal requires cautious framing, our multivariable model concurrently confirms a robust and independent association between IGA and accelerated cellular aging via LTL shortening in male adolescents. These collective findings suggest that individual mitochondrial genomic backgrounds may not act as direct, definitive causal drivers of addiction phenotypes, but rather play a subtle, modulatory role in behavioral addiction pathways by establishing an underlying biological vulnerability. Although these genetic associations remain preliminary and strictly associative, they provide intriguing, novel insights into the potential biological and molecular markers of excessive internet gaming behavior. These results substantially enhance our understanding of the biological correlates of IGA and may inform future longitudinal research aimed at identifying at-risk individuals and further validating the clinical utility of these complex, age-dependent genomic variations. 

## Figures and Tables

**Table 1:** Comparison of D-loop polymorphism and mtDNA copy number between the IGA and non-IGA groups (*n* = 206).

Variable			Non-IGA (*n* = 101)	IGA (*n* = 105)	*p*
			*n* (%) or M ± SD	*n* (%) or M ± SD	
mtDNA D-loop polymorphism	(C)_n_	6	3 (3.0)	1 (1.0)	0.040
		7	35 (34.7)	44 (41.9)	
		8	46 (45.5)	31 (29.5)	
		9	16 (15.8)	27 (25.7)	
		10	1 (1.0)	2 (1.9)	
	(CA)_n_	4	37 (36.6)	43 (41.0)	0.525
		5	62 (61.4)	60 (57.1)	
		6	2 (2.0)	2 (1.9)	
mtCN			25,313.28 ± 13,305.26	23,092.85 ± 13,442.17	0.247 ^b^

Note. The *p*-value was determined using the χ^2^ test, except for polymorphisms with a frequency of <5%, namely, (C)_6_, (C)_10_, and (CA)_6_. ^b^ Student’s *t*-test; (C)_n_, cytosine repeats; (CA)_n_, cytosine–adenosine repeats; M ± SD, mean ± standard deviation; mtCN, mitochondrial DNA copy number; mtDNA, mitochondrial DNA; IGA, internet gaming addiction.

**Table 2:** Comparison of D-loop polymorphisms and mtDNA copy number according to baseline characteristics (*n* = 196).

Variable	Category		mtDNA D-Loop Polymorphism							mtCN	
			(C)_n_				(CA)_n_				
			7	8	9		4	5			
		*n* (%)	*n* (%) or M ± SD			*p*	*n* (%) or M ± SD		*p*	M ± SD	*p*
Age (years)	15	28 (14.3)	17 (60.7)	7 (25.0)	4 (14.3)	0.141	9 (32.1)	19 (67.9)	0.363	26,369.05 ± 3720.56	0.156
	16	52 (26.5)	18 (34.6)	25 (48.1)	9 (17.3)		20 (38.5)	32 (61.5)		26,759.12 ± 1831.18	
	17	79 (40.3)	28 (35.4)	33 (41.8)	18 (22.8)		37 (46.8)	42 (53.2)		21,778.63 ± 3797.86	
	18	37 (18.9)	15 (40.5)	11 (29.7)	11 (29.7)		12 (32.4)	25 (67.6)		23,968.12 ± 3887.16	
Smoking	No	141 (71.9)	54 (38.3)	56 (39.7)	31 (22.0)	0.790	64 (45.4)	77 (54.6)	0.010	25,551.16 ± 3428.06	0.020
	Yes	55 (28.1)	24 (43.6)	20 (36.4)	11 (20.0)		14 (25.5)	41 (74.5)		20,625.92 ± 2727.76	
Alcohol	No	143 (73.0)	53 (37.1)	62 (43.4)	28 (19.6)	0.095	57 (39.9)	86 (60.1)	0.976	26,412.79 ± 3302.76	<0.001
consumption	Yes	53 (27.0)	25 (47.2)	14 (26.4)	14 (26.4)		21 (39.6)	32 (60.4)		18,115.30 ± 1734.48	
Sleep duration	≤6	133 (67.9)	53 (39.8)	53 (39.8)	27 (20.3)	0.832	52 (39.1)	81 (60.9)	0.772	24,716.54 ± 3657.63	0.407
(h/day)	>6	63 (32.1)	25 (39.7)	23 (36.5)	15 (23.8)		26 (41.3)	37 (58.7)		23,013.32 ± 2829.71	
mtCN			23,213.72 ± 12,405.33	26,040.77 ± 14,620.17	22,556.45 ± 12,710.49	0.289	26,438.62 ± 13,689.65	22,668.88 ± 13,026.51	0.053	-	-
LTL			166.51 ± 70.77	173.56 ± 78.89	156.81 ± 64.66	0.487	160.38 ± 74.68	171.64 ± 71.25	0.289	-	0.318

Note. (C)_6_, (C)_10_, and (CA)_6_ were excluded from the analysis because the frequency of these polymorphisms was <5%. (C)_n_, cytosine repeats; (CA)_n_, cytosine–adenosine repeats; M ± SD, mean ± standard deviation; mtCN, mitochondrial DNA copy number; mtDNA, mitochondrial DNA; LTL, leukocyte telomere length.

**Table 3:** Comparison of the frequency of mitochondrial D-loop (C)_n_ and (CA)_n_ between the IGA and non-IGA groups (*n* = 196).

mtDNA D-Loop		Non-IGA (*n* = 95)	IGA (*n* = 101)	*p*
Polymorphism		*n* (%)	*n* (%)	
(C)_n_	(C)_n_ = 7	34 (35.8)	44 (43.6)	0.266
	(C)_n_ ≠ 7	61 (64.2)	57 (56.4)	
	(C)_n_ = 8	45 (47.4)	31 (30.7)	0.017
	(C)_n_ ≠ 8	50 (52.6)	70 (69.3)	
	(C)_n_ = 9	16 (16.8)	26 (25.7)	0.129
	(C)_n_ ≠ 9	79 (83.2)	75 (74.3)	
(CA)_n_	(CA)_n_ = 4	36 (37.9)	42 (41.6)	0.598
	(CA)_n_ ≠ 4	59 (62.1)	59 (58.4)	
	(CA)_n_ = 5	59 (62.1)	59 (58.4)	0.598
	(CA)_n_ ≠ 5	36 (37.9)	42 (41.6)	

Note. (C)_6_, (C)_10_, and (CA)_6_ were excluded from the analysis because the frequency of these polymorphisms was <5%. (C)_n_, cytosine repeats; (CA)_n_, cytosine–adenosine repeats; mtDNA, mitochondrial DNA; IGA, internet gaming addiction.

**Table 4:** Multivariable logistic regression analysis of factors associated with internet gaming addiction (*n* = 196).

Variable	*B*	*SE*	*OR* (Exp(B))	95% *CI*	*p*
Constant	−0.561	0.218	1.753	–	0.101
Sleep duration	−0.655	0.316	0.519	0.280 to 0.965	0.038
(C)_8_ polymorphism	−0.746	0.302	0.474	0.262 to 0.858	0.014

Note. (C)_6_, (C)_10_, and (CA)_6_ were excluded from the analysis because the frequency of these polymorphisms was <5%. Abbreviations: *B*, regression coefficient; *SE*, standard error; *OR*, odds ratio; *CI*, confidence interval. Dummy coding: IGA (0 = Non-IGA, 1 = IGA); Sleep duration (0 = ≤6 h, 1 = >6 h); C_8_ (0 = absent, 1 = present).

**Table 5:** Comparison of mtDNA copy number and leukocyte telomere length (LTL) between the IGA and non-IGA groups according to mtDNA D-loop polymorphisms (*n* = 196).

mtDNA D-Loop Polymorphism		mtDNA Copy Number					LTL				
		Non-IGA		IGA		*p*	Non-IGA		IGA		*p*
		*n*	M ± SD	*n*	M ± SD		*n*	M ± SD	*n*	M ± SD	
(C)_n_	7	34	23,877.09 ± 10,986.93	44	22,701.12 ± 13,502.50	0.681	34	175.54 ± 69.92	44	159.52 ± 71.07	0.323
	8	45	27,837.35 ± 14,517.81	31	23,432.82 ± 14,607.49	0.199	45	198.69 ± 87.04	31	137.06 ± 46.00	0.001
	9	16	21,266.24 ± 13,576.14	26	23,350.42 ± 12,354.94	0.612	16	179.40 ± 53.05	26	142.91 ± 68.13	0.075
(CA)_n_	4	36	28,940.21 ± 12,842.82	42	24,294.39 ± 14,175.79	0.136	36	177.78 ± 79.37	42	145.95 ± 68.08	0.066
	5	59	23,100.24 ± 13,199.69	59	22,237.51 ± 12,949.72	0.721	59	193.25 ± 74.55	59	150.04 ± 61.09	0.001

Note. (C)_6_, (C)_10_, and (CA)_6_ were excluded from the analysis because the frequency of these polymorphisms was <5%. (C)_n_, cytosine repeats; (CA)_n_, cytosine–adenosine repeats; M ± SD, mean ± standard deviation; mtDNA, mitochondrial DNA; IGA, internet gaming addiction; LTL, leukocyte telomere length.

**Table 6:** Multivariable linear regression analysis of factors associated with leukocyte telomere length (*n* = 196).

Variable	*B*	*SE*	*β*	*t*	*p*	95% *CI*
(Constant)	184.291	12.104	–	15.226	<0.001	–
Smoking	−22.695	14.566	−0.150	−1.850	0.121	−51.427 to 6.037
Alcohol consumption	24.466	14.635	0.141	1.947	0.096	−4.403 to 53.335
Sleep duration	−19.709	10.826	−0.127	−1.820	0.070	−41.064 to 1.647
IGA	−40.180	10.250	−0.277	−3.920	<0.001	−60.400 to −19.960
(C)_8_	4.965	10.559	0.033	0.470	0.639	−15.864 to 25.793
(CA)_5_	12.853	10.481	0.087	1.226	0.222	−7.821 to 33.527

Note. (C)_6_, (C)_10_, and (CA)_6_ were excluded from the analysis because the frequency of these polymorphisms was <5%. Abbreviations: *B*, unstandardized coefficient; *SE*, standard error; *β*, standardized coefficient; *CI*, confidence interval; IGA, internet gaming addiction. Dummy coding: IGA (0 = Non-IGA, 1 = IGA); alcohol (0 = no, 1 = yes); smoking (0 = no, 1 = yes); CA5 (0 = absent, 1 = present); C_8_ (0 = absent, 1 = present); sleep duration (0 = ≤6 h, 1 = >6 h).

## Data Availability

The datasets presented in this article are not readily available in order to protect patient privacy. Requests to access the datasets should be directed to the corresponding author.

## Abbreviations

The following abbreviations are used in this manuscript:

(C)n	Cytosine repeats
(CA)n	Cytosine–adenosine repeats
DSM-5	Diagnostic and Statistical Manual of Mental Disorders, 5th Edition
D-loop	Mitochondrial displacement loop
HGB	Human β-globin
ICD-11	11th revision of the International Classification of Diseases
IGA	Internet gaming addiction
LTL	Leukocyte telomere length
mtCN	mtDNA copy number
mtDNA	Mitochondrial DNA
nDNA	Nuclear DNA
qPCR	Quantitative polymerase chain reaction
ROS	Reactive oxygen species
T/S	Telomere to single-copy gene ratio
WHO	World Health Organization
